# Comparative numerical and experimental evaluation of 3D-printed micromixers for biotechnological applications

**DOI:** 10.1038/s41598-026-68945-1

**Published:** 2026-08-28

**Authors:** Michael Mengele, Josef Zürn, Tanja Lochner, Ursula Weiß, Malte A. Peter, Janina Bahnemann, Christopher Heuer

**Affiliations:** 1 Institute of Physics, University of Augsburg, 86159 Augsburg, Germany; 2 Institute of Mathematics, University of Augsburg, 86159 Augsburg, Germany; 3 Centre for Advanced Analytics and Predictive Sciences (CAAPS), University of Augsburg, 86159 Augsburg, Germany

**Keywords:** 3D printing, Microfluidics, CFD, Mammalian cell culture, Shear sensitivity, Energy-dissipation rate, Biological techniques, Biotechnology, Engineering

## Abstract

Micromixers are essential in microfluidic-based biotechnology and diagnostics, where reproducible reactions and rapid mass transfer are crucial. Although many geometries exist, consistent comparison across dimensions, fabrication methods, and evaluation techniques remains challenging. In this study, we present a comparative analysis of six micromixer designs, focusing on mixing efficiency, pressure drop, shear stress, and the energy-dissipation rate. The devices were fabricated using high-definition 3D printing and studied experimentally and with high-resolution computational fluid dynamics (CFD) simulations. Tests covered a wide Reynolds-number range (Re = 0.1–200). At both ends of this range, most designs achieved adequate mixing. The greatest disparities emerged in the intermediate regime (Re = 1–50), with the Kenics geometry outperforming all other designs. Our grid-optimized CFD results matched experimental trends and quantified local shear and energy-dissipation rates. Live/dead assays using an industrially relevant Chinese hamster ovary (CHO) cell line showed unchanged viability immediately after mixing and during subsequent cultivation. By combining experimentally validated CFD predictions, systematic grid-optimized assessment, and biological validation within a single framework, this study establishes a consistent basis for comparing micromixer designs and selecting those that match desired combinations of mixing performance, pressure drop, volume, shear stress, and the energy-dissipation rate.

**Supplementary Information:**

The online version contains supplementary material available at https://doi.org/10.1038/s41598-026-68945-1.

## Introduction

Microfluidic technologies are gaining prominence in clinical and biotechnological contexts, from point-of-care diagnostics and environmental monitoring to cell culture and drug development. Their compact form factor, low reagent consumption, and compatibility with automated workflows make them attractive for both translational research and clinical implementation^1,2^. Microfluidic platforms have enabled rapid and sensitive analytical assays, including nucleic acid and protein quantification^3,4^, cell-based enrichment methods^5^, and extracellular vesicle detection^6^, thereby contributing to advances in personalized medicine^2^. In biotechnology, they provide highly controlled microenvironments for high-throughput drug screening, microbial or mammalian cell culture, and synthetic biology applications that exceed the capabilities of conventional systems^1^.

Efficient fluid mixing represents a key functional requirement in continuous-flow microfluidic systems, since operations such as reagent delivery^7,8^ and particle formation^9–13^ depend on rapid and reproducible homogenization under laminar-flow conditions^14^. In microchannels, flow is characterized by low Reynolds numbers, where viscous forces outweigh inertial contributions and turbulence does not develop. Under these conditions, mass transport is generally dominated by molecular diffusion, resulting in undesirably long characteristic mixing lengths and timescales^14^.

To overcome these limitations, specifically engineered channel geometries have been developed to introduce additional advective transport mechanisms that promote secondary flows and chaotic advection. In contrast to active micromixers, which rely on external energy input such as electric, magnetic, acoustic, or thermal fields, passive micromixers achieve mixing solely through geometric design and flow-induced effects. This makes them simpler to fabricate, easier to integrate, and more robust in continuous-flow operation^15^. Examples include Dean vortices in curved channels^16,17^, repeated lamination and stretching of fluid interfaces^18^, and split-and-recombine sequences^19^. These passive mixing strategies enhance the interfacial area between fluid streams and promote cross-sectional advection without relying on turbulence, thereby enabling efficient homogenization even under strictly laminar-flow conditions^14,20,21^. While these effects are typically enhanced by faster flows, the flow speed is often limited in practice, leading to the question of optimal channel geometry given the maximum possible Reynolds number.

Over the past two decades, a wide range of passive micromixer geometries based on repeated elements has been developed to exploit these advective transport mechanisms under laminar-flow conditions. Prominent examples include Tesla-type structures^22–25^, Kenics helical elements^26,27^, split-and-recombine (SAR) units^19,28–30^, staggered herringbone mixers^31^, Dean-vortex and spiral based geometries^17,32,33^, obstacle-based designs^34^, and expansion–contraction elements^18,35^. While many micromixer geometries demonstrate high mixing performance, results across studies are difficult to compare because they are obtained under widely differing conditions. Variations in channel dimensions, fabrication methods, Reynolds-number ranges, and evaluation techniques prevent direct comparison of reported mixing efficiencies. Moreover, pressure-drop data are not consistently reported, and quantitative assessments of shear stress or local energy-dissipation rates are less commonly included, further limiting cross-study comparability.

In addition to the lack of comparability between published geometries, methodological limitations further hinder a systematic assessment of micromixer performance. Many computational fluid dynamics (CFD) studies rely on coarse meshes or insufficiently strict mesh-convergence criteria, which may introduce numerical diffusion and lead to overestimated mixing efficiencies^36–38^. Reported mixing indices often differ in their mathematical definitions, spatial evaluation windows, or intensity-normalization strategies, resulting in inconsistencies even when nominally similar conditions are studied^15^. Together, these methodological discrepancies make it challenging to compare studies, validate simulations, or establish reliable performance benchmarks for micromixer designs.

The increasing availability of high-resolution 3D printing has made it possible to fabricate complex micromixer geometries with reproducible channel dimensions^39^. This enables side-by-side comparisons of fundamentally different designs under identical fabrication constraints, something that is difficult to achieve with traditional soft-lithography or multi-step microfabrication workflows. Although 3D-printed micromixers are becoming more common^26,39^, systematic comparisons of different geometries within a single experimental and computational framework remain limited. Most existing comparative studies are either purely numerical^40,41^ or based on soft-lithography^42,43^, which is largely restricted to planar designs. In each case, the assessment is confined to mixing and hydraulic performance.

These considerations are especially relevant in biotechnological applications, where micromixers are increasingly used in devices for gradient generation^44^, biosensing^45^, continuous cryoprotectant-cell mixing^46^, or nanoparticle formation^8^. In such contexts, mixing efficiency must be balanced with mechanical stress exposure, as mammalian cells—particularly suspension-adapted Chinese hamster ovary (CHO) cells—exhibit sensitivity to hydrodynamic stress. Elevated shear rates, local stress hotspots, and high energy-dissipation levels can impair viability, alter cell morphology, or affect downstream productivity^47^. Despite the relevance of these factors, quantitative assessments of local shear stress, energy-dissipation rates, and subsequent cellular responses are not routinely included in micromixer studies. As a result, the impact of common micromixer geometries on cell viability remains insufficiently characterized, and design choices are often made without quantitative information on cell-relevant hydrodynamic conditions.

To address these combined gaps, we present a systematic comparison of six 3D-printed passive micromixer geometries across a broad Reynolds-number range (0.1–200), covering regimes commonly encountered in microfluidic applications. Using experiments and CFD simulations, we assess mixing efficiency, pressure drop, shear stress, and local energy-dissipation rates under identical conditions. Numerical predictions are validated against experimental measurements and assessed for mesh convergence at several Reynolds numbers to avoid numerical artifacts. To assess cellular impact, CHO cell viability and viable cell density is analyzed immediately after passage through each mixer and during subsequent cultivation. By integrating experimental evaluation, CFD analysis, and cell-based assays, this work provides a unified framework for benchmarking micromixer performance and for selecting geometries suited to biotechnological and cell-related applications.

## Results and discussion

### Experimental mixing performance

**Fig. 1:**
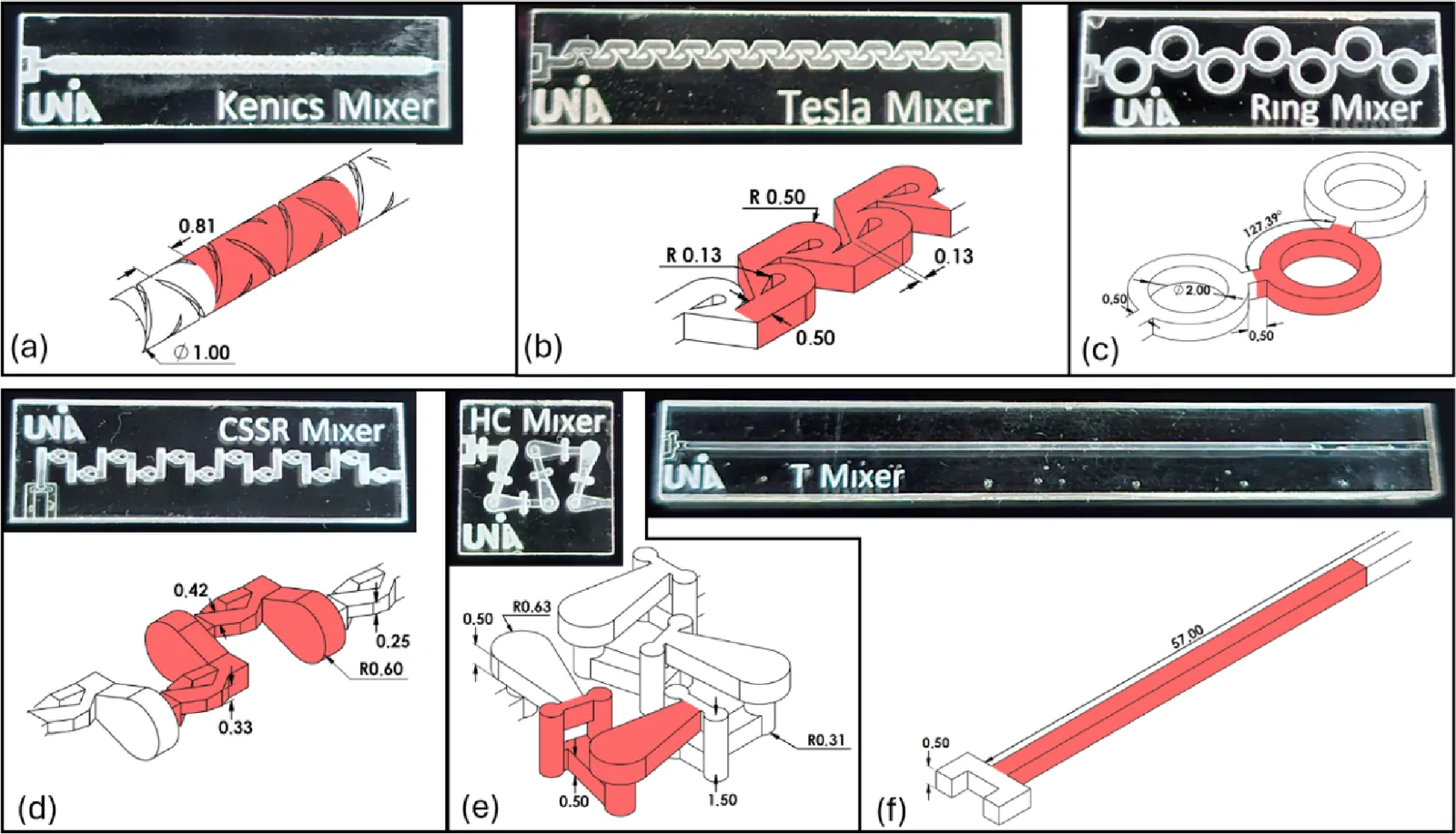
Overview of the six micromixer geometries: (**a**) Kenics, (**b**) Tesla, (**c**) DVBM, (**d**) CSSR, (**e**) HC, and (**f**) T-mixer. Photographs of the 3D-printed devices without HPLC-threaded inlets/outlets and the corresponding CAD models with dimensions (mm) are shown. The regions highlighted in red denote representative subvolumes, each accounting for approximately one sixth of the total internal mixer volume and defining the evaluation positions used for mixing analysis.

To compare different micromixing strategies systematically, six representative passive micromixer geometries were selected (Fig. 1). These include three widely studied high-performance designs: the Tesla mixer^25^, the Kenics mixer^48^, and the HC mixer^18^(a combination of H- and C-type elements). The Dean-vortex bifurcating mixer (DVBM)^16^, also referred to as the Ring mixer^49^, is included owing to its frequent use in nanoparticle-formulation workflows in biotechnological settings^12,13,50^. To extend this set of established geometries, the CSSR mixer combines two advection-enhancing features, the C-element used in the HC mixer and the splitting–stretching–recombination (SSR) structure introduced by Wang et al.^19^. A straight T-mixer serves as a baseline reference.

To enable comparison across all geometries, the internal channel volume was used as a common design parameter, and each mixer was standardized to an internal volume of approximately 15 µL. All designs shared an identical inlet geometry. As the repeating units of the different designs differ in size and shape, this volume-based standardization results in a different number of elements per mixer. Figure 1 shows photographs of the printed and post-processed mixers alongside their corresponding computer-aided design (CAD) representations, in which a representative subvolume corresponding to approximately one sixth of the total mixer volume is highlighted in red. Detailed geometrical dimensions of all micromixers are provided in Supplementary Fig. S1. Mixing performance was evaluated at the outlet of each of these subvolumes.

Surface roughness of the printed devices depended on the orientation relative to the build direction, with an arithmetic mean roughness of approximately 0.6 μm on the channel floor and ceiling and up to 9 μm on the side walls (Supplementary Figs. S2 and S3). Channel dimensions closely matched the nominal geometry, with a depth of 499.4 ± 1.2 μm and a width of 499.3 ± 10.5 μm against a nominal value of 500 μm (Supplementary Fig. S4).

A variety of experimental techniques has been used in the literature to quantify mixing in microfluidic systems, including pH-indicator reactions such as phenolphthalein^39^, passive colorimetric dyes^26,31^, and fluorescence-based assays^19,28,51,52^. pH-indicator dyes are inexpensive and provide well-defined color transitions, making them suitable for quantitative mixing analyses. In this study, Bromophenol Blue was selected; its transition from yellow-green to blue-violet provides strong visual contrast, allowing the progression of mixing between the two inlet streams to be clearly observed.

**Fig. 2:**
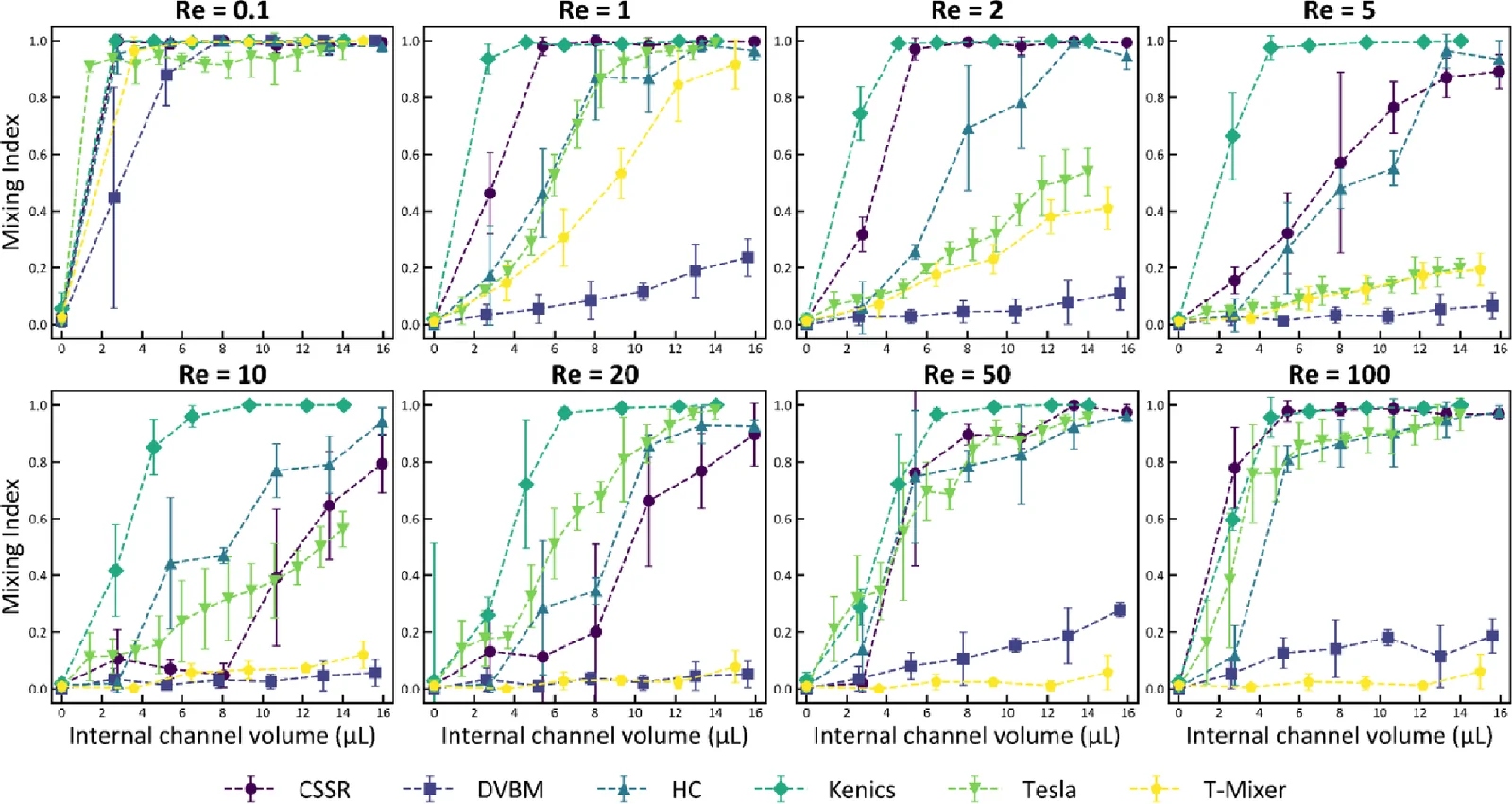
Mixing index evolution along the channel volume for six micromixer geometries (CSSR, DVBM, HC, Kenics, Tesla, T-mixer) measured at eight Reynolds numbers. Each of the eight panels displays the experimentally determined mixing indices for all six designs. Error bars correspond to the standard deviation of triplicate measurements.

Using this indicator, the mixing performance of all six micromixer designs was experimentally quantified, with representative images of the measurements provided in the Supplementary Information (Supplementary Figs. S5-S11). The Reynolds number was defined using the identical inlet cross-section shared by all six geometries, enabling direct comparison across the structurally distinct designs (see Methods). The mixing index (MI) is shown as a function of channel volume for Reynolds numbers ranging from 0.1 to 100 (Fig. 2), where MI = 0 indicates no mixing and MI = 1 corresponds to complete homogenization.

At Re = 0.1, mixing was driven mainly by diffusion, resulting in homogenization within a small internal channel volume (2–4 µL). Under these conditions, the mixing behavior of all geometries was nearly identical. At slightly higher Reynolds numbers (Re = 1–10), mixing performance decreased progressively and markedly for most designs. Diffusion becomes increasingly insufficient in this regime, while geometry-induced advection is not yet strong enough to compensate. As a result, at Re = 10 only the Kenics and HC mixers achieved complete mixing (MI > 0.9) within the available channel volume, whereas the CSSR mixer reached MI = 0.79. The Tesla mixer showed the most pronounced reduction in mixing efficiency among the high-performance designs, yielding substantially lower mixing indices than the Kenics, HC, and CSSR mixers. Notably, the DVBM exhibited an exceptionally low mixing index already at Re = 1 (with a mixing performance worse than that of the T-mixer), indicating that its bifurcating structure hinders diffusive transport.

As the Reynolds number increased further into the advection-dominated regime (Re ≈ 10–50), the Kenics mixer consistently achieved fast homogenization, requiring only small internal volumes to reach MI > 0.9. This reflects the efficiency of repeated splitting, rotation, and reorientation of fluid interfaces. The CSSR, HC, and Tesla mixers also showed progressively stronger mixing enhancement as inertial and geometry-induced secondary flows became more pronounced.

At even higher Reynolds numbers (Re ≥ 50), four designs—the Kenics, Tesla, HC, and CSSR mixers—reached high levels of homogenization across the tested volume range, whereas the DVBM and T-mixer did not. The limited mixing performance of the DVBM may appear at odds with its widespread use in nanoparticle production. At the channel dimensions used here, the Dean vortices required for efficient mixing appear not to be fully developed even at the highest flow rate tested, consistent with the low mixing indices observed across the entire Reynolds-number range. In nanoparticle production, ring mixers are operated at considerably higher flow rates^12,49^. In addition, their simple geometry is compatible with injection molding^49^ and avoids the high-resolution printing required for more intricate designs.

Across all conditions, the error bars represent the standard deviation of triplicate measurements. The observed variability was highest in the transitional mixing range (0.3 < MI < 0.8) between stable laminar flow and efficient mixing. In this range the mixing index responds steeply to small changes in flow conditions, so that minor variations between independently performed experiments, such as deviations in the prepared dye solutions or in the printed devices, translate into comparatively large differences in the measured mixing index. As mixing progressed and the signal became more homogeneous, this variability decreased substantially.

**Fig. 3:**
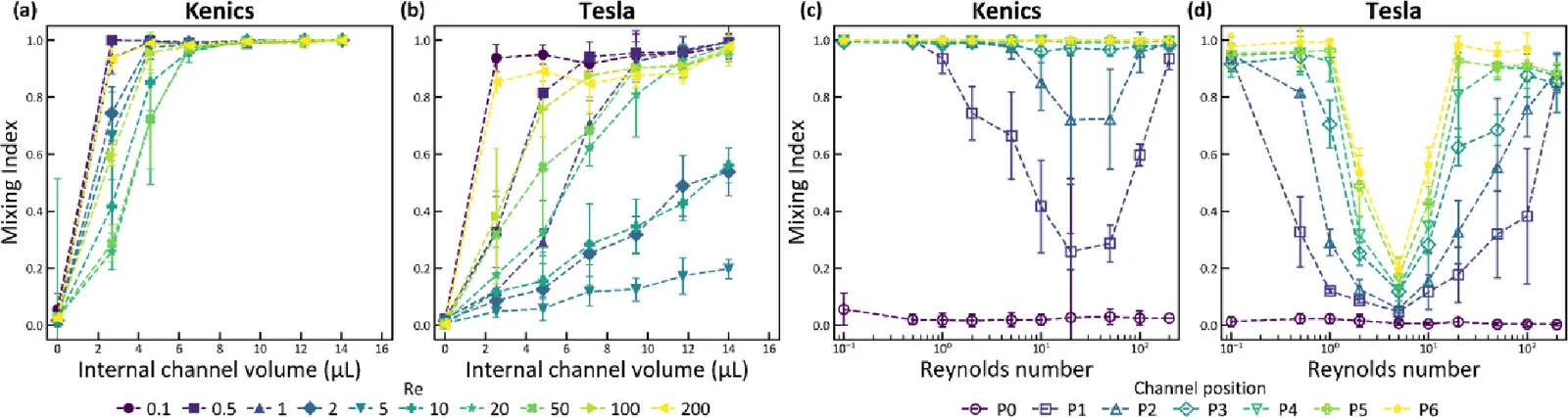
Experimental mixing index characterization of the Tesla (**a**, **c**) and Kenics (**b**, **d**) micromixers. (**a**, **b**) Mixing index as a function of channel volume for ten distinct flow rates (Reynolds numbers). (**c**, **d**) Mixing index as a function of Reynolds number, evaluated at predefined Regions of Interest (ROI) along the channel (P0 – P6). An example of the ROI definition is shown in the Supplementary Information (Supplementary Fig. S13). In all panels, error bars indicate the standard deviation from three independent measurements.

Figure 3 presents a focused comparison of the Tesla and Kenics geometries, which most clearly illustrate the contrasting mixing behavior discussed here. Equivalent data for the remaining four geometries, covering all flow rates up to Re = 200, are provided in the Supplementary Information (Supplementary Fig. S12). Although both mixers achieve high mixing efficiencies at larger Reynolds numbers, their performance diverges considerably in the intermediate regime. Volume-resolved mixing curves show that the Kenics mixer rapidly approaches full mixing for all tested Reynolds numbers, whereas the Tesla mixer exhibits a notable plateau at intermediate Reynolds numbers where mixing barely progresses. The logarithmic plots of mixing index versus Reynolds number further emphasize these trends: the Tesla mixer exhibits a distinct minimum around Re ≈ 5, while the Kenics mixer shows only a shallow decline around Re ≈ 50. The reduced performance of the Tesla mixer in the intermediate Reynolds-number regime arises from the absence of sufficiently developed recirculating backflow, which is characteristic of Tesla-type mixers at higher flow rates^22,24^. The formation of pronounced vortical structures requires stronger inertial effects and therefore occurs only at higher Reynolds numbers (see Supplementary Fig. S20). In the intermediate regime, diffusion remains the dominant transport mechanism, and no additional geometry-induced advection is present to enhance mixing significantly. Similar Reynolds-number-dependent mixing behavior has been reported previously for Tesla-type micromixers^24^. Together, these observations illustrate the strong dependence of micromixer performance on the interaction between geometry and flow regime and underscore the robustness of the Kenics mixer in transitional conditions.

### CFD simulation and mesh convergence study

CFD simulations were used to complement the experimental analysis by providing access to flow, shear, and transport quantities that cannot be measured experimentally at sufficient spatial resolution. All simulations were conducted using COMSOL Multiphysics^®^ v. 6.3^53^ under steady-state laminar flow conditions. To ensure the reliability of the numerical predictions, particular attention was paid to model formulation and to a systematic assessment of mesh dependence.

**Fig. 4:**
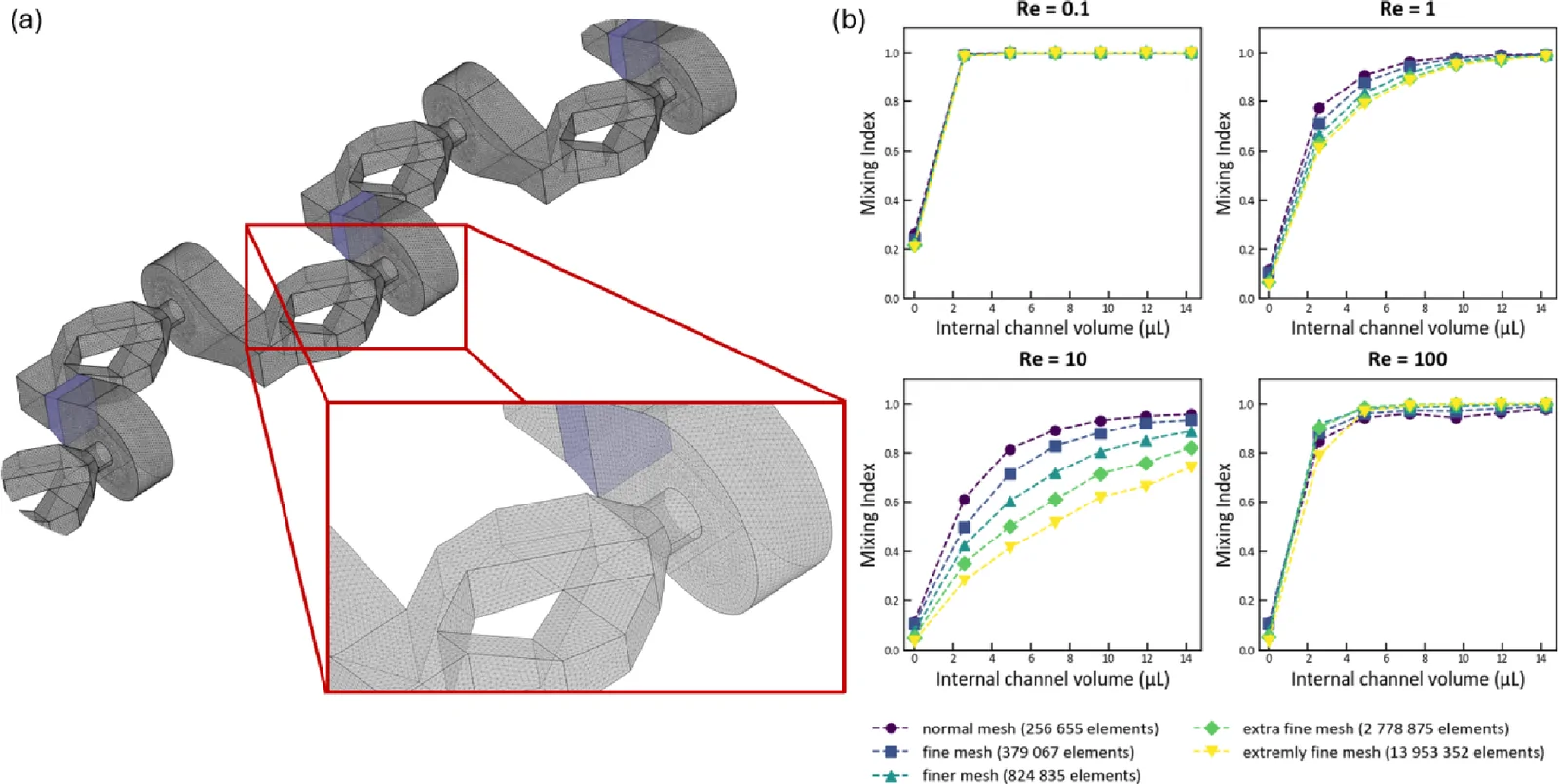
(**a**) COMSOL-generated finite-element mesh of the CSSR-mixer at the highest refinement level (extremely fine mesh, maximum element size: 34 μm), including an enlarged inset and highlighted regions of interest (ROI). (**b**) Mixing index curves for the CSSR mixer at four Reynolds numbers, with each panel showing the results obtained for all five mesh densities applied in the convergence study.

Figure 4a illustrates the finest discretization level considered in this study, shown here for the CSSR mixer, with a maximum element size of 34 μm. Preliminary simulations indicated that particular care is required when generating meshes in narrow geometric features that are considerably smaller than the main channel diameter, as these regions can induce rapid local changes in flow behavior. Consequently, the standardly generated meshes had to be adjusted to ensure sufficient resolution in these regions.

Panel 4b presents representative results of the mesh-convergence study for the CSSR mixer, showing the mixing index as a function of channel volume for four Reynolds numbers. Each panel compares five different mesh densities, ranging from adjusted COMSOL meshes labeled as “normal” to “extremely fine”.

The results show that at very low (Re = 0.1) and high Reynolds numbers (Re = 100), all mesh levels yield nearly identical mixing-index curves, indicating stable convergence at both ends of the considered velocity range. At Re = 1, the results exhibit a clear convergence trend with progressively finer meshes. In contrast, in the intermediate regime (Re = 10), the predicted mixing index shows a pronounced dependence on mesh resolution, with finer meshes producing noticeably flatter curve shapes and altered mixing-index predictions. This behavior suggests increased numerical sensitivity in the transitional regime, in which diffusion and geometry-induced advection contribute at comparable levels to mass transport.

This non-uniform convergence behavior indicates that micromixer simulations are particularly mesh sensitive in the transitional Reynolds-number range, where neither diffusion nor advection clearly dominates. Mesh-convergence studies performed solely at low or high Reynolds numbers, as commonly reported in the literature^30^, therefore allow only limited conclusions regarding numerical reliability in this range, and convergence should be assessed across flow regimes.

Numerical uncertainty was quantified for the CSSR mixer using the grid convergence index^54^. While negligible at Re = 0.1, 1 and 100, the uncertainty peaks at Re = 10 reaching a median of ± 0.18 and a maximum of ± 0.26 in mixing index across the seven evaluation positions (Supplementary Fig. S14). Outside the transitional regime, the numerical uncertainty is small enough for the simulations to be interpreted quantitatively. Within the transitional regime, quantitative statements rest on the experimental mixing indices. The shape of the mixing curves is nevertheless preserved across mesh levels; thus, the simulations still show qualitative trends. The simulations also remain the only source for quantities such as shear stress and energy-dissipation rate.

In addition to the mesh-convergence assessment, the influence of COMSOL’s built-in crosswind diffusion stabilization was evaluated. This consistent stabilization method is enabled by default and adds numerical diffusion to the momentum equations to aid convergence and to stabilize sharp shear layers that would otherwise require very fine mesh resolution^55^. Since even consistent stabilization can introduce artificial smoothing of the velocity field on finite meshes, which could in turn affect the predicted species transport, its influence on the present model was explicitly tested, both to assess the sensitivity of the results to the numerical scheme and to keep the model formulation as lean as possible. For the species transport interface, the default stabilization settings were retained. Supplementary Figure S15 shows the mixing index of the CSSR mixer at four Reynolds numbers, each for the five mesh densities used in the convergence study, with and without crosswind diffusion enabled. The curves with and without stabilization overlap closely under all conditions, indicating a negligible effect on the predicted mixing index.

Based on the convergence analysis and considerations of computational cost and comparability, all subsequent simulations of mixing behavior were performed using a uniform mesh with an element size of 30 μm and deactivated crosswind diffusion.

### Comparison of simulation and experimental results

**Fig. 5:**
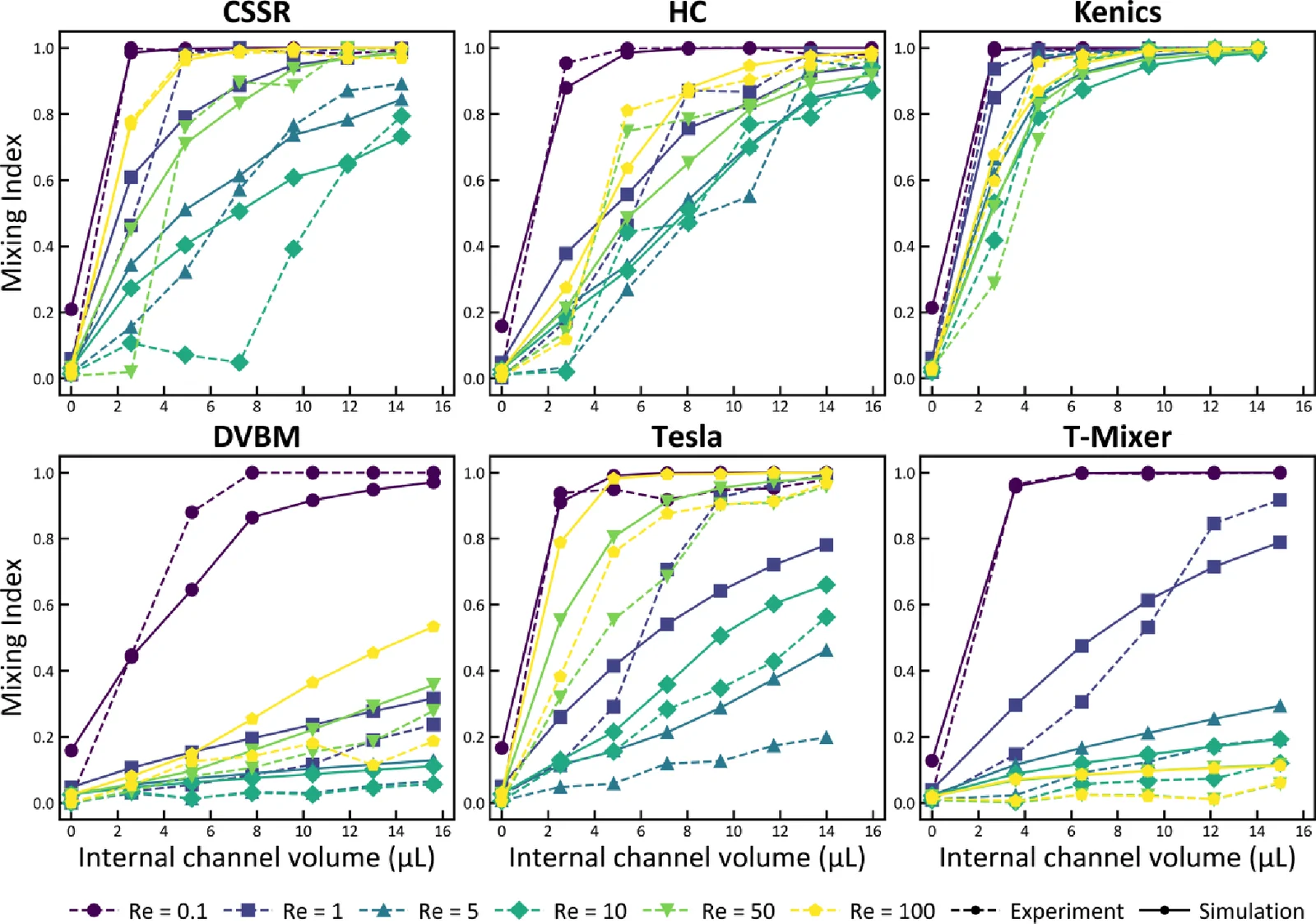
Comparison of experimental and simulated mixing-index profiles for all six micromixer geometries. For each mixer, the mixing index is plotted as a function of internal channel volume at six Reynolds numbers. Dashed lines indicate experimental measurements, and solid lines represent CFD predictions.

Figure 5 compares the experimentally measured mixing indices with the corresponding CFD predictions for all six micromixer geometries. Each plot shows the mixing index as a function of channel volume for six Reynolds numbers. Representative streamline plots of the simulated mixers are provided in Supplementary Figs. S16–S21. Across all geometries, the simulations reproduced the experimentally observed mixing progression closely. Accordingly, Lin’s concordance correlation coefficient – where a value of 1 indicates perfect agreement – ranges from 0.920 to 0.982 across the micromixer geometries (Supplementary Fig. S22). The mean difference between simulated and measured mixing index, resolved by Reynolds number and averaged over all evaluation positions, reveals where deviations occur (Supplementary Fig. S23). The Kenics, T-mixer and HC geometries deviated by at most 0.07 across the entire range, whereas the CSSR, DVBM and Tesla mixers reached deviations of up to 0.16. These larger deviations were not confined to a fixed Reynolds-number range but occurred at Re = 10 for the CSSR mixer, at Re = 100 for the DVBM and at Re = 5 for the Tesla mixer. In each case, this corresponds to the transitional regime of the respective geometry, in which diffusive and advective transport contribute to a similar extent to mass transfer and the numerical sensitivity identified in the convergence study is greatest. In the Tesla mixer, these larger deviations persisted even at higher Reynolds numbers.

The experimental measurements also exhibited substantial variability in the weakly mixed regime, which contributed to deviations between the two datasets. Differences in data acquisition methods may also contribute to these deviations. In the experiment, the mixing indices are derived from color analysis at the mixer surface within defined Regions of Interest (ROIs), whereas the simulations provide full three-dimensional volumetric data of the ROIs. The simulations further assume smooth channel walls and therefore do not account for the roughness of the printed side walls (Supplementary Figs. S2 and S3). Because roughness would be expected to enhance rather than suppress mixing in the experiments, it cannot explain why simulated mixing indices occasionally exceed measured ones. Overall, the CFD predictions matched the experimental data closely for most geometries and conditions, while caution is required in the transitional regime of each design, where numerical approximation errors are largest.

### Hydrodynamic characteristics

To complement the mixing analysis, CFD simulations verified by the mesh-convergence analysis were used to quantify hydrodynamic metrics that cannot be measured experimentally with sufficient spatial resolution, including pressure drop, shear stress, and local energy-dissipation rates. In microfluidic systems, pressure drop represents a key design constraint, as it directly determines the required pumping power, limits achievable flow rates, and affects the scalability and integration of devices into larger fluidic workflows. High pressure losses can restrict parallelization, increase mechanical stress on system components, and complicate operation in pump-limited or cell-handling applications, making pressure efficiency a critical metric alongside mixing performance^56^. This aspect is particularly critical for miniaturized, self-contained benchtop systems, where pumping is typically battery-powered and the available energy is limited^57^.

To capture both the global hydraulic demand and the effectiveness with which pressure input is converted into mixing, pressure drops were evaluated across the full device length as well as at the location where a mixing index of 0.9 is reached. The corresponding values for all mixer geometries are summarized in Table 1. At Re = 100, the Tesla and CSSR mixers exhibit the highest overall pressure drops. In the Tesla mixer, the channel geometry induces localized backflow, as shown in Supplementary Fig. S20, while the CSSR mixer incorporates a narrow constriction that strongly accelerates the flow, resulting in substantial viscous losses and an increased hydraulic resistance. Although these geometric features lead to elevated hydraulic resistance, both mixers achieve 90% mixing within the smallest internal volumes, indicating efficient inertial mixing at the expense of an increased pressure drop. At Re = 10, only the HC and Kenics mixers reach a mixing index of 0.9. In this regime, the HC mixer operates at a slightly lower pressure drop, whereas the Kenics mixer reaches the target mixing index within a substantially smaller channel volume of approximately 59%.

**Table 1:** Quantification of hydraulic performance through simulation for the six micromixer designs. The table reports both the overall pressure drop across the complete device and the pressure drop and corresponding internal channel volume at the location where the mixing index exceeds 90% for the first time.

	T-mixer	DVBM	Tesla mixer	Kenics mixer	HC mixer	CSSR mixer
Total device Re = 100						
Volume (µL)	15	15.61	13.92	14.04	15.94	14.24
∆P (Pa)	1355	671	8490	2401	1498	8440
MI > 90% Re = 100						
Number of elements	–	–	4/12	6/14	4/6	2/6
Partial volume (µL)	–	–	4.83	6.49	10.68	4.91
∆P (Pa)	–	–	3012	1131	1013	2868
MI > 90% Re = 10						
Number of elements	–	–	–	9/14	6/6	–
Partial volume (µL)	–	–	–	9.34	15.94	–
∆P (Pa)	–	–	–	81	73	–

To assess the hydrodynamic conditions experienced by cells within the micromixers, shear stress and energy-dissipation rate (EDR) were evaluated from the simulated velocity field. Both metrics are derived from the symmetric part of the velocity-gradient tensor and therefore exhibit similar qualitative spatial patterns. The magnitude of shear stress provides an intuitive indicator of regions with steep velocity gradients, for example in narrow channels or near geometric constrictions (see Supplementary Fig. S24). Shear stress represents a tangential force per unit area acting parallel to a surface within the fluid and is widely used to characterize hydrodynamic forces acting on cells, particularly in studies of adherent cell cultures^58^.

However, complex flow fields of real fluids, such as those encountered in micromixers in biotechnology, may additionally involve extensional deformation and mixed shear-extensional kinematics arising from spatial variations in the velocity field. In such cases, shear stress alone does not fully describe the overall intensity of hydrodynamic deformation experienced by suspended cells. The energy-dissipation rate, in contrast, quantifies the rate at which mechanical energy is converted into viscous losses within the fluid and therefore integrates the effects of both shear and extensional deformation. Because it provides a scalar measure of the overall deformation intensity within the flow field, EDR is widely used to characterize hydrodynamic stress environments in bioprocessing systems and studies of suspension cell cultivation^47^, and is computed here according to Eq. (12).

**Fig. 6:**
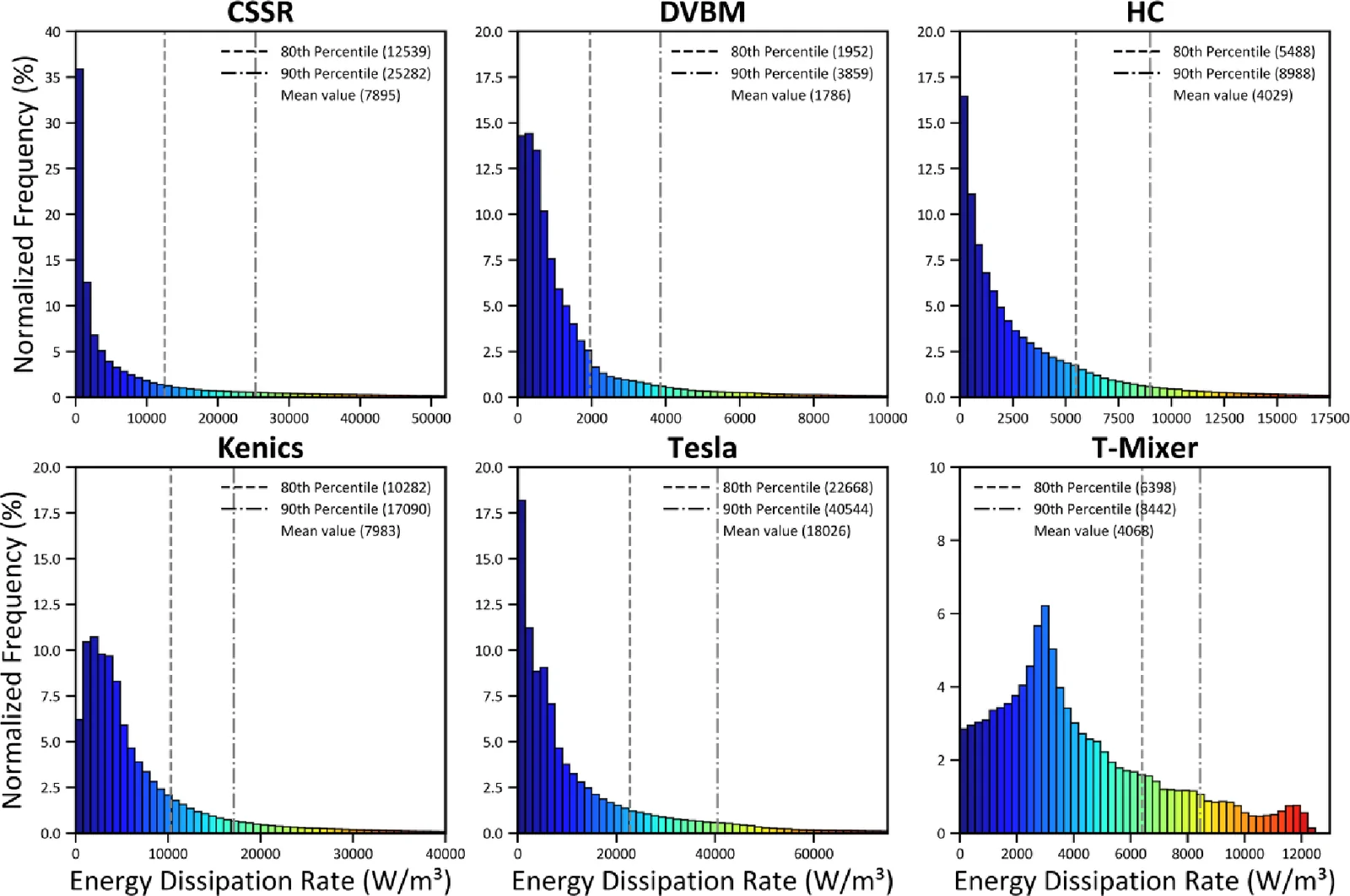
Energy dissipation rate histograms obtained from CFD simulations for each of the six micromixer designs, evaluated over one mixing element. Dashed vertical markers denote the 80th and 90th percentile thresholds of the local dissipation values.

Because the energy-dissipation rate provides a more representative measure of hydrodynamic deformation, only EDR is shown here, although both quantities were computed for each mixer using the smallest representative mixing element to ensure that no geometric repetition influenced the distributions. To enable a meaningful comparison of hydrodynamic loads, all values were extracted at Reynolds number 100, where geometry-induced advective effects are pronounced across all designs.

The resulting energy-dissipation-rate distributions, shown in Fig. 6, reveal pronounced quantitative differences between the six micromixer geometries, while the corresponding shear-stress distributions (Supplementary Fig. S25) exhibit qualitatively similar spatial patterns. In all cases, most of the flow domain is characterized by moderate energy-dissipation rates, while only a small fraction experiences elevated local values. To characterize both the typical and extreme hydrodynamic loading, mean values as well as the 80th and 90th percentiles of the local EDR distributions were evaluated for one mixing element of each design.

At Re = 100, the CSSR, Kenics and Tesla mixers exhibit the highest overall dissipation levels, with mean EDR values exceeding several thousand W m⁻³ and the 90th percentile extending well into the 10⁴ W m⁻³ range. These elevated values originate from the narrow constriction and the associated strong accelerations of the flow, which generate intense secondary flows.

The HC and T-mixer geometries occupy an intermediate regime. While their mean EDR values remain substantially below those of the CSSR, Kenics and Tesla mixers, the upper percentiles indicate the presence of localized regions with increased dissipation. Notably, the T-mixer exhibits relatively high dissipation levels despite poor mixing. Standardized to the same internal volume but lacking internal structures, it realizes this volume as a long, narrow channel, where high mean velocities produce steep wall-normal velocity gradients without corresponding mixing enhancement. In contrast, the HC mixer combines effective mixing with moderate dissipation, distributing fluid deformation over the flow domain rather than concentrating it in localized high-gradient regions. The DVBM exhibits the lowest mean and percentile EDR values among all designs, consistent with its smoother flow paths and reduced geometric forcing.

The maximum values of local energy-dissipation rates observed in this study reach approximately 8 × 10^4^ W m^-3^. These values remain well below the dissipation levels reported by J. J. Chalmers for lethal hydrodynamic responses of suspended CHO cells, which are typically on the order of 10^6^–10^7^ W m^-3^^47^. In this context, the present results indicate that efficient micromixing can be achieved without approaching dissipation levels associated with mechanically damaging conditions.

### Cell response to mixing

**Fig. 7:**
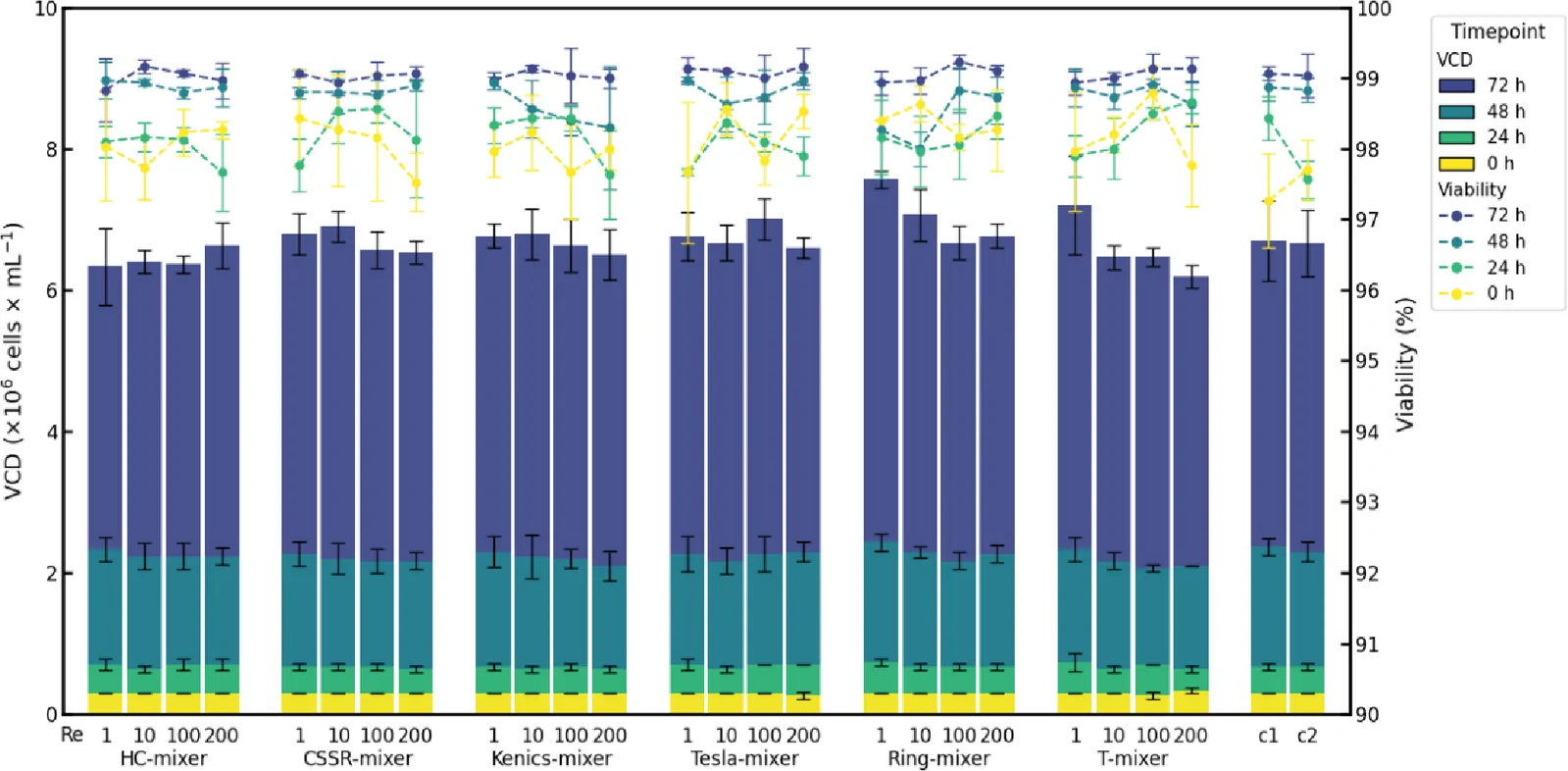
Viable-cell density (VCD) and viability of CHO cells after passage through six micromixer geometries and two controls (c1, c2). For each mixer, VCD values at 0 h, 24 h, 48 h, and 72 h are shown for four Reynolds numbers (Re = 1, 10, 100, 200) as depth-ordered, partially overlapping bars. Corresponding viabilities at the same instances in time are displayed as line plots. Error bars represent the standard deviation of three independent measurements.

Although the hydrodynamic analysis suggests that the applied mixing conditions are well below levels associated with mechanical cell damage, an experimental assessment was performed to verify that effects not represented in the simulations, such as channel wall roughness (Supplementary Figs. S2 and S3) or the biocompatibility of the printing material, do not influence cell viability under realistic operating conditions. To assess the impact of the hydrodynamic conditions generated within the six micromixer geometries, CHO suspension cells were exposed once to each mixer at Reynolds numbers of 1, 10, 100, and 200. Reynolds numbers above 200 were not examined, as the corresponding flow rates already exceed those required for the intended cell-handling applications, for example microfluidic transfection^8^, and the same upper limit was used in the dye-based mixing experiments. Figure 7 summarizes the resulting viable cell densities (VCD) and viabilities, determined using a trypan blue exclusion assay, measured immediately after passage (0 h) and during subsequent cultivation over 24, 48, and 72 h. The bars for each condition are shown in a layered format, which leads to partial overlap of individual columns, and error bars represent the standard deviation of triplicate measurements.

Across all mixers and flow conditions, VCD and viability were comparable to those of untreated controls. Immediately after exposure, a slight decrease in viability of 1–2% was detected. However, the cells fully recovered during the 72 h cultivation period, and no mixer or Reynolds-number condition led to sustained viability loss or impaired cell growth.

These results indicate that, despite geometric constrictions, localized shear-stress elevations, and the measured roughness of the channel side walls, a single pass through any of the tested mixers does not measurably affect CHO cell viability or subsequent growth under the examined operating conditions.

## Conclusion

The combined experimental and computational analysis highlights clear differences in performance among the investigated micromixer geometries. At very low Reynolds numbers, where diffusion dominates, all designs behaved similarly. The largest differences emerged in the intermediate regime (Re = 1 to 50), where diffusion is no longer sufficient and geometry-induced advection is not yet fully established. Only the Kenics and HC mixers maintained high mixing indices throughout this range, whereas the Tesla mixer showed a pronounced minimum and recovered only at higher Reynolds numbers. The DVBM and T-mixer did not reach complete mixing at any condition tested above Re = 1.

Hydrodynamic loading did not scale with mixing performance. The maximum energy-dissipation rates observed here reached approximately 8 × 10⁴ W m⁻³, one to two orders of magnitude below levels reported to damage suspended mammalian cells. Consistent with this, a single pass through any of the tested geometries did not measurably reduce CHO cell viability or viable cell density over 72 h of subsequent cultivation. As this assessment was limited to a single CHO suspension cell line, sensitivity to hydrodynamic stress should be evaluated separately for other cell types.

The mesh-convergence study showed non-uniform convergence behavior: predictions at very low and high Reynolds numbers were largely insensitive to mesh density, whereas the intermediate regime remained mesh-dependent even at the finest resolution used, so that quantitative statements in this range rest on the experimental data rather than on the simulations. More generally, this indicates that convergence assessments performed at a single, favorable operating point may overstate the reliability of micromixer simulations, and that the intermediate regime merits dedicated numerical investigation.

These results do not support a single figure of merit for micromixer selection, since mixing efficiency, pressure drop, hydrodynamic loading, footprint, and manufacturability are weighted differently across applications and can act in opposite directions. They do, however, allow application-oriented choices to be made explicit. Where efficient mixing is required across a wide range of flow rates, for example in continuous processing at variable throughput, the Kenics geometry was the most robust of the designs tested. Where back pressure is limiting, as in integrated lab-on-a-chip devices, the HC geometry offered comparable mixing at lower hydraulic demand. Where devices are to be mass-produced by injection molding, the planar Tesla and DVBM geometries are preferable, since both avoid complex internal structures. Both require high flow rates to mix effectively, with the DVBM requiring considerably higher ones than the Tesla mixer. Rather than prescribing a single design, the framework presented here provides the quantitative basis for weighting such trade-offs according to the requirements of a given application.

## Materials and methods

### Micromixer designs and device fabrication

The micromixer geometries were created and adapted in SolidWorks 2024 (Dassault Systèmes, Vélizy-Villacoublay, France) and fabricated using a Projet MJP 2500 Plus multijet printer (3D Systems, Rock Hill, USA). Printing was carried out in the high-definition mode, providing a nominal layer height of 32 μm and an in-plane resolution of 800 × 900 dpi^59^. VisiJet M2S-HT90 was used as the build material, while the wax-based VisiJet M2 SUP served as the support material.

After printing, the parts were incubated at -21 °C for approximately 10 min to facilitate removal from the build platform. Most of the wax-based support material was dissolved in a 15-minute steam bath (EasyClean unit, 3D Systems), followed by an ultrasonic oil-bath treatment (65 °C, 15 min) (Elmasonic S 30 H, Elma, Singen, Germany) using paraffin oil (Carl Roth GmbH, Karlsruhe, Germany). Residual support material within internal features was removed through sequential ultrasonic cleaning steps, first in a detergent solution and subsequently in deionized water. The channels were additionally flushed using a syringe with oil, detergent solution, and finally deionized water until no residues remained. Following post-processing, the devices received a transparent surface coating (Luxaprint^®^ shellac, Detax, Ettlingen, Germany) to enhance optical clarity for the colorimetric mixing experiments. For the cell viability assays, no coating was applied; instead, the mixers were sterilized (121 °C, 20 min) by autoclaving prior to use.

### Surface characterization and dimensional accuracy

Surface roughness was characterized on a reference part printed and post-processed as described above, since the resulting surface quality is governed by the printing process and material rather than by the specific device geometry.

Three mutually orthogonal build planes were examined: the XY plane, corresponding to the top and bottom surfaces, and the two side walls, ZX and ZY, along the two axes of the build platform. Areal topography was recorded on a Keyence VK-X4000 laser-scanning microscope (Keyence, Osaka, Japan) operated in combined laser-confocal and white-light mode, which yields a co-registered optical image and height map of the same area. A 20×/0.46 plan objective was used throughout. Each analyzed area was a 2 × 2 stitched image, and three spatially separated areas were recorded per plane to sample within-surface variability.

Height data were read directly from the binary VK4 height block using Python. Within each area, six profiles were extracted along a fixed 25/50/75% grid, three in each image direction, giving 18 profiles per plane. Each profile was tilt-corrected by subtracting a least-squares straight line and cropped centrally to a common evaluation length of 990 μm. Profiles were evaluated without additional filtering, and roughness is reported as Ra and Rz.

Dimensional accuracy was assessed on a reference part printed with open channels of 500 μm nominal width and depth, since the enclosed channels of the mixer devices are not optically accessible. Three channels were measured at three spatially separated areas each. For each area, a median cross-section was computed from the profiles of the areal height map. Heights were referenced to a line fitted through the flat regions on either side of the channel, so that the surface adjacent to the channel defines the zero level. Channel depth was taken as the difference between this reference and the median height of the channel floor. Channel width was evaluated at 5% of the channel depth, since near-vertical side walls exceed the maximum slope resolvable with the objective used and are therefore not reliably reconstructed.

### Mixing-performance experiments

The acidic Bromophenol Blue solution (AnalaR Normapur^®^ ACS grade, VWR International, Leuven, Belgium) was prepared by dissolving the dye at 1 g L^-1^ in distilled water (pH 3.0). The alkaline counterpart was adjusted to pH 9.7 by adding sodium hydroxide to distilled water, ensuring that the indicator transition occurs at 50% mixing.

Mixing experiments were conducted using the two prepared solutions delivered by a syringe pump (AL-1000, World Precision Instruments, Sarasota, USA) equipped with a custom holder for two syringes, and imaged using a digital light microscope operated in transmission mode (VHX-6000, Keyence, Osaka, Japan). The syringes were connected to the micromixer chips via PTFE tubing (inner diameter of 0.5 mm, 1/16″ outer diameter), using 1/4″-28 threaded connectors (see Supplementary Fig. S26). Flow rates in the range of 3 to 6018 µL min^-1^ were evaluated, corresponding to Reynolds numbers between 0.1 and 200. Because the internal channel cross-section varies continuously and non-uniformly along the flow path within each geometry, no single local dimension can serve as a representative, comparable length scale across the six markedly different designs. The Reynolds number was therefore calculated in the identical square inlet section shared by all mixers, providing a common, geometry-independent reference for direct comparison across designs, according to:

1 $$\:\mathrm{R}\mathrm{e}=\:\frac{\rho\:vL}{\mu\:},$$

where *ρ* = 997 kg m^-3^ is the density of water, $\:v\:$the average inlet velocity, *µ* = 1 mPa s the dynamic viscosity of water, and *L* the hydraulic diameter of the inlet channel. For a rectangular cross-section with width *w* and height *h*, the hydraulic diameter was computed as^39^

2 $$\:L=\:\frac{2wh}{w+h}.$$

Mixing quality was quantified by evaluating the blue-channel intensity within predefined Regions of Interest (ROIs). ROIs covered the full channel width and were kept identical for all measurements of a given mixer. For normalization, two reference ROIs were defined: one unmixed inlet state and one fully mixed state obtained under steady-state flow (see Supplementary Fig. S13). These regions provided the mean intensities *I*_0_ (unmixed) and *I*_∞_ (fully mixed) and the corresponding standard deviations *σ*_0_ and *σ*_∞_.

The Mixing Index MI_*i*_ for each ROI *i* was calculated as:

3 $$\:\mathrm{M}{\mathrm{I}}_{i}=\mathrm{M}{\mathrm{I}}_{I,i}\cdot\:\mathrm{M}{\mathrm{I}}_{\sigma,i}=\frac{\mid\:{I}_{i}-{I}_{0}\mid\:}{\mid\:{I}_{{\infty\:}}-{I}_{0}\mid\:}\cdot\:\frac{{\sigma\:}_{0}-{\sigma\:}_{i}}{{\sigma\:}_{0}-{\sigma\:}_{{\infty\:}}}.$$

The first term reflects how closely the local mean intensity approaches the fully mixed reference, whereas the second captures the reduction of spatial intensity variations. MI reaches unity only when both the target mean intensity and the required level of spatial homogenization are achieved, preventing artificially high values caused by incidental intensity matches or locally uniform yet unmixed regions.

Each condition was measured in triplicate, where each replicate represented an independently performed experiment on a different day using freshly printed mixer devices and newly prepared dye solutions. Mixing indices are reported as mean ± standard deviation.

### CFD setup and mesh-convergence analysis

The mixer geometries were imported into COMSOL Multiphysics^®^ 6.3^53^(COMSOL AB, Stockholm, Sweden) as SolidWorks part files (“.SLDPRT”) and discretized using the laminar flow and transport of diluted species interfaces. All simulations assumed an incompressible Newtonian fluid, and the material properties were set to those of water at 20 °C (*ρ* = 998 kg m^-3^, *µ* = 1.01 mPa s), with a molecular diffusion coefficient of 1 × 10^-9^ m^2^ s^-^¹ applied for the transported species. Steady-state simulations were used for all cases, which is appropriate for the low-to-intermediate Reynolds numbers considered in this study^27,28^, and consistent with the absence of any experimentally observable unsteady behavior. Thus, the numerical approximation of the solution to the steady-state incompressible Navier–Stokes equations was found, which determined the advection velocity used in the subsequent numerical approximation of the solution to a linear advection–diffusion equation.

At the two inlets, fully developed velocity profiles were imposed for the flow, and fixed species concentrations of 1 mol m⁻³ and 2 mol m⁻³ were prescribed for the respective incoming streams. At the outlet, a zero-gauge pressure condition was applied for the flow, combined with an outflow condition for the species transport. All channel walls were treated as no-slip boundaries for the flow and no-flux boundaries for the species transport.

All geometries were discretized using unstructured tetrahedral elements. For the mesh-convergence study, COMSOL’s predefined mesh levels ranging from normal to extremely fine were used as reference. Based on these analyses, a standardized mesh configuration was selected for all subsequent simulations to ensure comparability across geometries. The final meshes were generated with a maximum element size of 30 μm and a maximum element growth rate of 1.05, without applying corner refinement. Depending on the mixer geometry, the resulting meshes comprised approximately 9 to 17 million elements (6 to 17 million degrees of freedom). For the Tesla mixer, an additional local refinement region with a minimum element size of 8 μm was introduced at the constriction to resolve steep velocity gradients adequately.

To balance numerical accuracy and computational cost, first-order Lagrange elements (P1–P1) were employed for velocity and pressure. Streamline diffusion was applied as consistent stabilization to ensure numerical stability. Crosswind diffusion, which is enabled by default in COMSOL, was deactivated after dedicated test simulations confirmed that it had a negligible effect on the predicted mixing index across all Reynolds numbers and mesh densities (see Supplementary Fig. S15), thereby avoiding unnecessary numerical diffusion and keeping the model formulation as lean as possible. For the Transport of Diluted Species, second-order Lagrange elements were used for species concentration to improve the resolution of concentration gradients. For this interface, streamline and crosswind diffusion were retained as stabilization.

Mesh-convergence analysis was performed on a representative geometry (the CSSR mixer) at four Reynolds numbers (Re = 0.1, 1, 10, and 100). The mixing index was used as the primary convergence metric and was evaluated in the same ROI as in the experiments. The mixing index (MI) was calculated using the following equation^15^:

4 $$\:\mathrm{M}\mathrm{I}=1-\:\sqrt{\frac{{\sigma\:}_{m}^{2}}{{\sigma\:}_{\mathrm{m}\mathrm{a}\mathrm{x}}^{2}}}\:,$$

Where

5 $$\:{\sigma\:}_{m\:}^{2}=\:\frac{1}{N}\:\sum\:_{i=1}^{N}{\left({c}_{i}-\:{c}_{\mathrm{a}\mathrm{v}}\right)}^{2},$$

6 $$\:{c}_{\mathrm{a}\mathrm{v}}=\:\frac{1}{N}\sum\:_{i=1}^{N}{c}_{i},$$

where *c*_*i*_ is the species concentration at discretization node *i*, $\:{c}_{\mathrm{a}\mathrm{v}}\:$is the mean concentration in the ROI, and *σ*_max_ is the standard deviation of a completely unmixed state (50:50 inlet condition). For each mesh level and flow condition, steady-state solutions for the velocity and concentration fields were computed, and the mixing index was evaluated at the same axial sampling volumes (ROIs) used in the experiments. The resulting MI-curves were compared across mesh levels to assess numerical sensitivity and determine the minimum refinement level required to achieve mesh-independent predictions.

The discretization uncertainty of the simulated mixing index was quantified for the CSSR mixer following the procedure of Celik et al.^54^, using the three finest of the mesh levels described above. Representative cell sizes were calculated as h = (V/N)^1/3^ from the nominal channel volume, giving refinement ratios of 1.71 and 1.50, both above the recommended minimum of 1.3. The observed order of convergence was determined separately for each combination of Reynolds number and evaluation position, and the numerical uncertainty was calculated with a safety factor of 1.25. In contrast to the relative formulation given by Celik et al., the uncertainty is reported in absolute units of the mixing index, since the mixing index is bounded between 0 and 1. At 8 of the 28 evaluated points, at which the convergence behavior was oscillatory or degenerate or the observed order fell outside the range 0.5 to 3.0, a conservative safety factor of 3.0 was applied instead.

Shear stress and energy-dissipation rates were computed directly from the velocity-gradient tensor $\:\nabla\:\boldsymbol{U}$ obtained from the CFD simulations. For an incompressible Newtonian fluid, the viscous shear stress tensor ***τ*** is defined as^60^.

7 $$\:\boldsymbol{\tau\:}=2\mu\:\boldsymbol{S},$$

where *µ* is the dynamic viscosity of the fluid and ***S*** is the strain-rate tensor. The strain-rate tensor is defined as the symmetric part of the velocity-gradient tensor^61^

8 $$\:\nabla\:\boldsymbol{U}=\:\frac{1}{2}\left(\nabla\:\boldsymbol{U}+{\left(\nabla\:\boldsymbol{U}\right)}^{\mathrm{T}}\right)\:+\:\frac{1}{2}\left(\nabla\:\boldsymbol{U}-{\left(\nabla\:\boldsymbol{U}\right)}^{\mathrm{T}}\right),$$

where the second term represents the antisymmetric part of the velocity gradient, corresponding to rotational velocity. Since rotational velocity does not contribute to viscous deformation, only the symmetric part is retained^60,62^,

9 $$\:\boldsymbol{S}=\frac{1}{2}\left(\nabla\:\boldsymbol{U}+{\left(\nabla\:\boldsymbol{U}\right)}^{\mathrm{T}}\right),$$

To evaluate the magnitude of the shear stress, the corresponding scalar shear rate *γ* was computed. The local shear rate was determined from the second invariant of the strain-rate tensor^63^,

10 $$\:\gamma\:=\:\sqrt{2\boldsymbol{S}\::\boldsymbol{S}},$$

This leads to the scalar relation for the magnitudes^64^

11 $$\:\tau\:=\mu\:\gamma\:$$

The local energy-dissipation rate (EDR) ε was computed from the same strain-rate tensor according to^62^

12 $$\:\epsilon\:=\:\boldsymbol{\tau\:}\::\nabla\:\boldsymbol{U}=2\mu\:\:\boldsymbol{S}\::\boldsymbol{S}=\mu\:{\gamma\:}^{2}.$$

Both quantities were extracted in post-processing from the CFD velocity field using COMSOL’s built-in variables *τ* = spf.mu*spf.sr and *ε* = spf.mu*spf.sr^2. Values were evaluated within the smallest representative mixing element of each geometry and at Reynolds number 100 to enable consistent comparison across designs.

### Assessment of CHO cell tolerance to hydrodynamic stress

For the stress-exposure experiments, a CHO-K1 suspension culture (cell line obtained from the Institute of Technical Chemistry, Leibniz University Hannover, Germany) was harvested and adjusted to a concentration of 3.0 × 10^7^ cells mL^-1^. Cell suspensions were pumped through each micromixer once using the same dual-syringe setup as in the mixing experiments, operated inside a sterile laminar-flow hood. Flow rates were selected to correspond to Reynolds numbers of 1, 10, 100, and 200. Immediately after passage, an aliquot was collected to determine the “0 h” viability. The remaining sample was transferred into fresh medium and cultivated for 72 h under standard conditions. Each replicate was measured in a randomized order of mixer geometries. The experiments were performed in two batches of three mixers, and at the end of each batch a control sample was collected under identical handling conditions but without passage through a micromixer.

Cells were inoculated at 0.3 × 10^6^ cells mL^-1^ in CHO-TF medium (Xell AG, Bielefeld, Germany) supplemented with 8 mM L-glutamine (Biowest, Nuaillé, France) and 0.1% Penicillin-Streptomycin (100X) (VWR chemicals, Radnor, USA). Cultures were incubated at 37 °C in a humidified atmosphere containing 5% CO_2_ using a New Brunswick S41i incubator (Eppendorf, Hamburg, Germany). The cells were cultivated in 15 mL bioreactor tubes (TubeSpin^®^ Bioreactor 15, TPP, Trasadingen, Switzerland) equipped with ventilated screw caps, agitated at 155 rpm, and operated at a working volume of 6 mL.

Cell density and viability were quantified at 0, 24, 48, and 72 h using an automated cell counter (Vi-CELL BLU, Beckman Coulter, Brea, USA) based on trypan blue exclusion. Each condition was measured in triplicate, where each replicate corresponded to an independently performed experiment conducted on a different day and culture passage. Viability values are reported as mean ± standard deviation.

## Supplementary Information

Below is the link to the electronic supplementary material.


Supplementary Material 1


## Data Availability

The data generated in this study are available in the Zenodo repository at https://doi.org/10.5281/zenodo.21533708 and will be made publicly accessible upon publication of this article.
