# Supplementary material for: Comparative numerical and experimental evaluation of 3D-printed micromixers for biotechnological applications
Source: Sci Rep. 2026 Aug 28;16:27109. doi: 10.1038/s41598-026-68945-1 (PMC13524954; doi:10.1038/s41598-026-68945-1)
Supplement: Supplementary file 1 [file 41598_2026_68945_MOESM1_ESM.docx]

**Supplementary Information**

**Comparative numerical and experimental evaluation of 3D-printed micromixers for biotechnological applications**

Michael Mengele^1,⸸^, Josef Zürn^1,⸸^, Tanja Lochner^2^, Ursula Weiß^3^, Malte A. Peter^2,3^, Janina Bahnemann^1,3^, and Christopher Heuer^1,3*^

^1^Institute of Physics, University of Augsburg, Augsburg, 86159, Germany
^2^Institute of Mathematics, University of Augsburg, Augsburg, 86159, Germany
^3^Centre for Advanced Analytics and Predictive Sciences (CAAPS), University of Augsburg, Augsburg, 86159, Germany
^*^Christopher.heuer@uni-a.de
^⸸^these authors contributed equally to this work

**Figure S1**. Technical drawings and dimensions of the micromixer geometries. (a) CSSR, (b) HC, (c) Tesla, (d) Kenics, (e) DVBM, and (f) T-Mixer. Detailed dimensions are specified in millimeters. Internal channels lengths were selected to fit the resolution of the MJP 2500 Plus.

**Figure S2.** Optical images and corresponding height maps of the three build planes. Left column: white-light images; right column: laser-confocal height maps of the same area. (a, b) XY plane, (c, d) ZX side wall, (e, f) ZY side wall. One of the three areas recorded per plane is shown. Axes are given in build coordinates, with x and y the in-plane print directions and z the build direction.

**Figure S3.** Height profiles of the three build planes along both image directions. (a) XY plane along x print direction, (b) XY plane along y print direction, (c) ZX plane along x, (d) ZX plane along z, (e) ZY plane along y, (f) ZY plane along z. Each panel shows the nine profiles of one plane and direction: three areas per sample, with three profiles per area.

**Figure S4**. Dimensional accuracy of the printed channels of 500 µm nominal width and depth. (a) Cross-sections of three channels, recorded at three separate areas per channel, with the surface adjacent to the channel taken as the zero reference (dashed line). (b) Channel depth and (c) channel width for each channel and area. Width was evaluated at 5 % of the channel depth, since the near-vertical side walls exceed the maximum slope resolvable with the objective used and are therefore not reliably reconstructed. Horizontal bars give the mean of the three areas per channel with the standard deviation, and the dashed line marks the nominal dimension of 500 µm.

**Figure S6.** Microscopic images of the Ring mixer recorded at different Reynolds numbers. The corresponding Reynolds number (Re) is shown in the upper left corner of each subpanel in a black box. The flow direction is from left to right. All images were taken using identical microscope settings to ensure comparability.

**Figure S5.** Microscopic images of the HC mixer recorded at different Reynolds numbers. The corresponding Reynolds number (Re) is shown in the upper left corner of each subpanel in a black box. The flow direction is from left to right. All images were taken using identical microscope settings to ensure comparability.

**Figure S8.** Microscopic images of the CSSR mixer recorded at different Reynolds numbers. The corresponding Reynolds number (Re) is shown in the upper right corner of each subpanel in a black box. The flow direction is from left to right. All images were taken using identical microscope settings to ensure comparability.

**Figure S7.** Microscopic images of the Tesla mixer recorded at different Reynolds numbers. The corresponding Reynolds number (Re) is shown in the bottom right corner of each subpanel in a black box. The flow direction is from left to right. All images were taken using identical microscope settings to ensure comparability.

**Figure S9.** Microscopic images of the T - mixer recorded at different Reynolds numbers. The corresponding Reynolds number (Re) is shown in the upper right corner of each subpanel in a black box. The flow direction is from left to right. All images were taken using identical microscope settings to ensure comparability.

**Figure S11.** Microscopy images of the Kenics micromixer recorded at a constant Reynolds number (Re = 20). Panels (a–f) correspond to mixers containing 2, 4, 6, 9, 12, and 14 mixing elements, respectively. Quantitative evaluation within the helical mixing section is hindered by optical distortion caused by the curved channel walls; therefore, the mixer lengths were selected such that each device terminates in a straight outlet region with a square cross-section, enabling reliable image analysis. For all mixer length (a–f), the complete velocity range under investigation was measured (not shown).

**Figure S10.** Microscopic images of the Kenics mixer recorded at different Reynolds numbers. The corresponding Reynolds number (Re) is shown in the upper left corner of each subpanel in a black box. The flow direction is from left to right. All images were taken using identical microscope settings to ensure comparability.

**Figure S13.** Microscopy image of the HC micromixer with the evaluated regions of interest (ROIs) indicated in red. The inlet reference intensity (I₀) is determined at the channel entrance, while the fully mixed reference intensity (I_∞_) is obtained at the outlet region downstream of the mixing structure.

**Figure S12.** Experimental mixing index characterization of the CSSR (a,c), DVBM (b,d), HC (e, g) and T- (f,h) micromixers. (a,b, e, f) Mixing index as a function of channel volume for ten distinct flow rates (Reynolds numbers). (c,d, g, h) Mixing index as a function of Reynolds number, evaluated at predefined Regions of Interest (ROI) along the channel (P0 – P6). In all panels, error bars indicate the standard deviation from three independent measurements.

**Figure S14.** Numerical uncertainty of the simulated mixing index for the CSSR mixer. Each panel shows the mixing index as a function of internal channel volume at the indicated Reynolds number. Filled symbols denote the values obtained on the finest mesh, and the shaded band the associated numerical uncertainty U_num_ estimated from the grid convergence index (GCI) and expressed in units of the mixing index. Open red symbols show the experimentally determined mixing indices, with error bars indicating the standard deviation of triplicate measurements.

**Figure S15.** Mesh convergence study of the mixing index for the CSSR mixer at four Reynolds numbers, each shown for five mesh densities (normal, fine, finer, extra fine, extremely fine). Red curves show simulations without crosswind; black curves show simulations with crosswind diffusion activated. At all four Reynolds numbers, the red and black curves overlap closely, indicating that the presence of crosswind has little to no effect on the mixing index.

**Figure S17.** COMSOL Multiphysics simulation results showing streamline plots of the concentration field within the HC mixer at different Reynolds numbers (Re = 0.1, 5, 10, and 100). The color scale represents the normalized species concentration, illustrating the evolution of mixing along the channel with increasing flow inertia.

**Figure S16.** COMSOL Multiphysics simulation results showing streamline plots of the concentration field within the CSSR mixer at different Reynolds numbers (Re = 0.1, 5, 10, and 100). The color scale represents the normalized species concentration, illustrating the evolution of mixing along the channel with increasing flow inertia.

**Figure S19.** COMSOL Multiphysics simulation results showing streamline plots of the concentration field within the Kenics mixer at different Reynolds numbers (Re = 0.1, 5, 10, and 100). The color scale represents the normalized species concentration, illustrating the evolution of mixing along the channel with increasing flow inertia.

**Figure S18.** COMSOL Multiphysics simulation results showing streamline plots of the concentration field within the Ring mixer at different Reynolds numbers (Re = 0.1, 5, 10, and 100). The color scale represents the normalized species concentration, illustrating the evolution of mixing along the channel with increasing flow inertia.

**Figure S21.** COMSOL Multiphysics simulation results showing streamline plots of the concentration field within the T-mixer at different Reynolds numbers (Re = 0.1, 5, 10, and 100). The color scale represents the normalized species concentration, illustrating the evolution of mixing along the channel with increasing flow inertia.

**Figure S20.** COMSOL Multiphysics simulation results showing streamline plots of the concentration field within the Tesla mixer at different Reynolds numbers (Re = 0.1, 5, 10, and 100). The color scale represents the normalized species concentration, illustrating the evolution of mixing along the channel with increasing flow inertia.

**Figure S23.** Deviation between simulated and experimentally determined mixing indices for the six micromixer geometries averaged over all evaluation positions. (a) Mean difference MI_sim_− MI_exp_ plottet against Reynolds number. Dashed lines connect values of the same geometry and serve to guide the eye, and the horizontal line marks perfect agreement. (b) The same differences shown for each geometry and Reynols number. The values give the signed difference, whereas the color scale represents its magnitude.

**Figure S22.** Agreement between simulated and experimentally determined mixing indices for the six micromixer geometries. Each panel shows the simulated mixing index (MI_sim_) plotted against the corresponding experimental value (MI_exp_) for one geometry: (a) CSSR, (b) DVBM, (c) HC, (d) Kenics, (e) Tesla, (f) T-mixer. Each panel contains between 36 and 42 values covering all Reynolds numbers and evaluation positions. Symbol shape and color denote the Reynolds number. The solid line indicates perfect agreement (1:1) and the shaded band a deviation of ±0.15 in mixing index. Agreement is quantified by Lin's concordance correlation coefficient (CCC), which measures the deviation of paired values from the 1:1 line and therefore accounts for both correlation and systematic offset; values range from −1 to 1, with 1 indicating perfect agreement.

**Figure S24.** COMSOL Multiphysics showing a slice plot of the shear stress distribution within the HC micromixer. The plot displays the shear stress magnitude evaluated at all Gauss integration points, with the color scale (Pa) indicating the local stress intensity throughout the channel and mixing structures.

**Figure S25.** Shear-stress distributions in terms of normalized frequency, i.e. number of recordings of the particular shear stress normalized by total number of recordings, obtained from CFD simulations for each of the six micromixer designs, evaluated over one mixing element. Dashed vertical markers denote the 80th and 90th percentile thresholds of local shear-stress values.

**Figure S26**. Top: Schematic overview of the experimental setup consisting of a syringe pump supplying the microfluidic device mounted on a digital microscope stage. Bottom: Detailed view of the microfluidic chip illustrating the inlet streams. Sodium hydroxide (NaOH) and bromophenol blue solutions are introduced through separate inlets and flow into the main channel, where mixing and reaction occur.
